# Factors associated with posttraumatic stress disorder and depression in war-survivors displaced in Croatia

**DOI:** 10.3325/cmj.2011.52.709

**Published:** 2011-12

**Authors:** Marina Letica-Crepulja, Ebru Salcioglu, Tanja Frančišković, Metin Basoglu

**Affiliations:** 1 Regional Psychotrauma Centre Rijeka and Department of Psychological medicine, Psychiatric Clinic, Clinical Hospital Centre Rijeka, Rijeka, Croatia; 2 Trauma Studies Unit, Institute of Psychiatry, King’s College, University of London, London, UK; 3 Istanbul Center for Behavior Research and Therapy, Istanbul, Turkey; 4 Department for Psychiatry and Psychological Medicine, University of Rijeka School of Medicine, Rijeka, Croatia

## Abstract

**Aim:**

To examine the role of perceived stressfulness of trauma exposure and economic, social, occupational, educational, and familial adaptation after trauma in posttraumatic stress disorder (PTSD) and depression in displaced war survivors.

**Methods:**

A cross-sectional survey was conducted between March 2000 and July 2002 with a sample of 173 internally displaced persons or refugees and 167 matched controls in Croatia. Clinical measures included Structured Clinical Interview for DSM-IV and Clinician-Administered PTSD Scale.

**Results:**

Displaced war survivors reported the exposure to a mean ± standard deviation of 13.1 ± 8.3 war stressors, including combat, torture, serious injury, death of close persons, and loss of property. Compared to controls, they reported higher rates of marked to severe impact of war on family (16.2% vs 51.6%), social (7.2% vs 43.5%), economic (12.6% vs 55.4%), occupational (1.8% vs 15.9%), and educational (2.4% vs 8.8%) adaptation. In two logistic regression analyses, the strongest predictor of PTSD and depression was high level of perceived distress during trauma exposure. PTSD but not depression was associated with economic, social, occupational, educational, and familial adaptation after trauma.

**Conclusion:**

Displaced survivors who experienced multiple war events perceived greater negative impact of war on their life domains compared to individuals who lived in a war setting but had no trauma exposure. The most important determinant of psychological outcomes was perceived stressfulness of war stressors. Although post-trauma adaptation in different life spheres had an impact, its effect was not robust and consistent across disorders. These findings suggest that it would be effective to use a trauma-focused approach in rehabilitation of war survivors.

Armed conflicts, wars, and associated displacement affect large numbers of people in the world (1). According to the United Nations High Commissioner of Refugees (UNHCR) (2), at the end of 2010 the number of forcibly displaced people in the world was 43.7 million. Some war survivors are displaced within the borders of their countries as internally displaced persons, while others are displaced to other countries as refugees. In addition to war-related stressor events they have been through, such displaced war survivors are believed to experience enduring contextual stressors, including socioeconomic disadvantage and poverty, changes in family structure and functioning, loss of social support, lack of access to education, overcrowded housing, hostility and racism, acculturation difficulties, marginalization and isolation, and cultural bereavement (3-7). These stressors are claimed to cause general psychological distress (8), but there are few studies that examined their contribution to mental health while controlling for the impact of other potentially important variables.

The most common mental health outcome of exposure to war-related traumatic stressors is posttraumatic stress disorder (PTSD) and depression (9). In a meta-analysis of 181 surveys comprising 81 866 refugees and other conflict-affected persons from 40 countries, the prevalence rate of PTSD across all surveys was 30.6% and that of depression was 30.8% (1). In this study, the factors that showed strong association with PTSD were cumulative exposure to traumatic events, time since conflict, and assessed level of political terror, while the factors associated with depression were cumulative exposure to traumatic events, time since trauma, torture, and residency status. An important factor that this meta-analysis did not control for (due to lack of data) was psychological processes during trauma exposure, including emotional reactions, perception of life threat, or dissociation during trauma, which have been determined to be the strongest predictors of PTSD (10-14). Indeed, in a series of studies involving war and torture survivors, distress and loss of control during exposure to traumatic stressors (9,15-19) emerged as the strongest predictors of PTSD when the effects of the number of trauma events (cumulative exposure) were statistically controlled for in a regression analysis.

Some studies identified older age (20,21), female gender (3,21), and psychiatric history and current illness (22) as predictors of traumatic stress reactions but it is not possible to reach a conclusion about these associations because these studies rarely took into account the effects of trauma exposure characteristics.

Political violence and terror during the 1991-1995 war in Croatia and the 1992-1995 war in Bosnia and Herzegovina were categorized as the highest level on the Political Terror Scale (23). Four years of conflict resulted in the displacement of almost 900 000 Croatian citizens of all ethnicities inside and outside the country. Serb secession in central and eastern parts of the country caused an internal displacement in Croatia of over 550 000 ethnic Croats. The war that erupted in Bosnia and Herzegovina in April 1992 resulted in a large population movement. Over the course of the conflict, Croatia accepted 403 000 refugees from the neighboring country (24). The total number of displaced war survivors in Croatia at the end of 1992 was more than 10% of the total Croatian population (25). In this study, we examined the factors associated with PTSD and depression in displaced war survivors in Croatia. Our aim was to determine the role of economic, social, occupational, educational, and familial adaptation after trauma on PTSD and depression while controlling for the effects of psychological processes during trauma exposure as well as demographic and personal history characteristics. Specifically, we hypothesized that 1) displaced war survivors who experienced multiple war-related potentially traumatic events would report greater negative impact on economic, social, occupational, educational, and familial adaptation compared to a control group of individuals who, despite living in a war setting, had no personal exposure to potentially traumatic events; 2) PTSD and depression would be most strongly associated with perceived stressfulness or uncontrollability of war-related traumatic stressors; and 3) economic, social, occupational, educational, and family adaptation in the aftermath of exposure to traumatic incidents would be an independent but weaker predictor of PTSD and depression.

## Methods

### Sampling procedures

Our research was part of a large survey conducted between March 2000 and July 2002. Details of the study methodology were presented in the main report (9). Briefly, a cross-sectional survey was conducted with a population-based sample of 1358 war survivors who had experienced at least 1 war-related stressor (combat, torture, internal displacement, refugee experience, siege, and/or aerial bombardment) from 4 sites in former Yugoslavia (Belgrade, Serbia and Montenegro; Rijeka, Croatia; Sarajevo, Bosnia-Herzegovina; and Banja Luka, Republic of Srpska, Bosnia-Herzegovina), accessed through linkage sampling. For the purposes of this study, from the total sample we selected 173 internally displaced persons and refugees (displaced war survivors hereafter) living in the region around Rijeka, Croatia. The internally displaced persons were mostly Croats from the Vukovar region. Inclusion criteria were age 18 through 65 years, literacy, absence of past or present psychotic illness, and written consent for participation in the study. Target sampling (26) was used to ensure adequate representation of survivor group of interest. Sampling bias in the targeted group was minimized as much as possible by using linkage sampling and snowballing (27). This strategy involved tracing and contacting survivors in the community through key informants who were asked to make a list of their friends or acquaintances with an experience of a particular index stressor, disregarding any available information on their psychological status. These survivors were then contacted and invited to participate in the study. Once the interview was completed, each survivor was asked to list all friends or acquaintances with a similar trauma experience. This “snowballing” process continued until the targeted sample size for a particular index stressor was achieved. The study design included a control group (n = 167) matched on a 1:1 basis with the community participants on sex, age, and education. Although most control participants were exposed to war scenes on television and/or had relatives or friends who had experienced war events, they had no direct exposure to war stressors.

### Measures

Psychiatric outcome was assessed with the Structured Clinical Interview for the Diagnostic and Statistical Manual of Mental Disorders (DSM-IV) (SCID-I/NP, version 2) (28). Assessment of current and lifetime PTSD was made with the Clinician-Administered PTSD Scale (CAPS) (29). Demographic, trauma characteristics, and direct social impact of war and displacement on economic, social, family, occupational, and educational functioning were assessed with the Semi-Structured Interview for Survivors of War (SISOW) (9), which was modified from the Semi-Structured Interview for Survivors of Torture (30) used in previous studies. The SISOW includes an Exposure to War Stressors Scale (EWSS) that elicits information on 54 war-related stressor events (eg, experience of combat, torture, witnessing atrocities, internal or external displacement, loss of close ones, etc) and perceived distress and uncontrollability associated with each event. Measurement included a Global Distress Rating (0 = not at all distressing, 4 = extremely distressing) and a Global Sense of Control Rating (0 = completely in control and 4 = not at all in control/entirely helpless) to assess displaced war survivors’ overall perceived distress or loss of control during exposure to such war stressors. The SISOW also provided 5 variables assessing the impact of trauma on economic, social, family, occupational, and educational life (0 = no impact at all, 4 = extremely severe impact). These variables reflected the cumulative negative impact of all traumatic events identified by the EWSS on various life spheres by comparing pre-war status with the present. In this assessment, interviewers distinguished as much as possible the impact of the traumatic stressors from the impact of PTSD on various life spheres. Specifically, the assessors were instructed to identify the trauma event responsible for the impact in a particular life sphere considering the nature of the latter (eg, physical disability, loss of job or social status, loss of educational opportunities, death in or separation from the family) and not to evaluate the negative aspect of PTSD symptoms (eg, on marital or family adaptation or work functioning).

### Interviews

The interviews were conducted by 5 trained professionals in Rijeka, Croatia. The interviews were evaluated for validity of diagnoses, rapport, adherence to the assessment protocol for each instrument, and overall quality of assessment. The concordance rates (kappa values) were 0.81 for PTSD, 0.84 for depression, and ranged from 0.83 to 0.99 for SISOW items in the overall sample (9). The interviews were usually completed in 1 or 2 sessions. Written informed consent was obtained for all study procedures. Strict confidentiality was emphasized. All study participants were offered an interview fee of US $10 and those in need of help were offered psychiatric treatment.

### Data analyses

Data were analyzed with SPSS, version 14.0 (SPSS Inc., Chicago, IL, USA). Sample characteristics were examined with frequency and descriptive statistics. Displaced war survivors were compared to matched controls on study variables of interest to produce evidence in support of the validity of the economic, social, family, occupational, and educational adaptation after trauma using McNemar test for related samples and paired *t* tests. Pearson correlation was used to examine the intercorrelations among impact of trauma on economic, social, family, occupational, and educational adaptation variables. As most of these variables were highly intercorrelated a composite measure representing global impact of trauma on life was computed for use in prediction analyses to avoid problems of multicollinearity. Two logistic regression analyses examined the factors related to current PTSD and current major depressive episode. Logistic regression allows one to predict a discrete outcome such as disease/no disease from a set of independent variables that may be continuous, discrete, dichotomous, or a mix (31). As in multiple regression analysis, the independent variables can be correlated with one another to varying degrees and the analysis takes into account these correlations in predicting the outcome. The goal of logistic regression is to correctly predict the category of outcome for individual cases using the most parsimonious model. To accomplish this goal, a model is created that includes all predictor variables that are useful in predicting the response variable. Since logistic regression calculates the probability or success over the probability of failure, the results of the analysis are in the form of an odds ratio. A Wald test was used to test the statistical significance of each coefficient of predictor variable (β). In this study, a total of 11 independent variables were entered in the logistic regression analyses, which included age, sex (1 = male, 2 = female), marital status (0 = single, 1 = married), education (1 = primary school, 2 = secondary school, 3 = high school, 4 = university/post-graduate), income level (1 = low, 4 = very good), personal and/or family history of psychiatric illness (0 = no, 1 = yes), time since last trauma event (months), global distress (0 = not at all distressing, 4 = extremely distressing) and control during trauma (0 = completely in control and 4 = not at all in control/entirely helpless) ratings, and the composite measure of global impact of trauma on life (total score on 5 variables ranging 0 to 20).

## Results

### Sample characteristics

The socio-demographic characteristics of the sample are given as web-extra material. There was a balanced distribution of male and female participants in the sample. The majority of the displaced war survivors were Croats (n = 148, 85.5%) and Catholics (n = 136, 78.6%). The mean ± standard deviation time since the most distressing traumatic event was 9.0 ± 1.6 years. Participants were exposed to a mean of 13.1 ± 8.3 war-related stressors. Some of these stressors included combat experience (n = 41, 23.7%), prisoner of war experience (n = 34, 19.7%), torture (n = 43, 24.9%), serious injury (n = 10, 5.8%), death of close persons (n = 83, 48.0%), exposure to aerial bombardment (n = 82, 47.4%), and sudden loss of property (n = 123, 71.1%). About 70% of the participants reported severe/extremely severe distress, while 94% reported slight to extreme loss of control during exposure to such traumatic stressors. Sixty-one participants (35.5%) had lifetime PTSD, 39 (22.5%) had current PTSD, 22 (12.7%) had current depression, 20 (11.6%) had past depression, 16 (9.2%) had alcohol abuse, and 10 (5.8%) had dysthymic disorder. Rates of the other psychiatric diagnoses were lower than 3%.

The matching procedure resulted in groups similar in sex, age, education, and marital status (Table 1). There was no difference between the groups in reported rates of personal and family history of psychiatric illness. As would be expected, displaced war survivors reported significantly greater number of war stressors that they had been exposed to. The two groups also differed in terms of income level. Compared to controls, displaced war survivors had significantly higher rates of PTSD (0.6% vs 22.8%, *P* < 0.001) and depression (2.4% vs 12.0%, *P* < 0.001). Among displaced war survivors, 51.6%, 43.5%, and 55.4% reported marked to severe negative impact on their family, social, and economic adaptation, respectively (Table 2). A comparison of the survivors and controls revealed marked differences in family, social, economic, occupational, and educational adaptation after trauma (all *P*’s significant at 0.001 level). The latter findings supported the validity of the scale.

**Table 1:** Comparison of displaced war survivors and matched controls on demographic, personal history, and trauma variables

	No. (%) of displaced war survivors (n = 167)	No. (%) of controls (n = 167)	McNemar test
Sex:			
men	74 (44.3)	74 (44.3)	*P* = 1.00*^†^
women	93 (55.7)	93 (55.7)	
Marital status:			
married	64 (38.3)	64 (38.3)	χ^2^ = 0.0, *P* = 1.0*
single/widowed/divorced	103 (61.7)	103 (61.7)	
Psychiatric illness before war	2 (1.2)	5 (3.0)	*P* = 0.45*^†^
Psychiatric illness in the family	46 (27.5)	41 (24.6)	χ^2^ = 0.25, *P* = 0.61*
Posttraumatic stress disorder	38 (22.8)	1 (0.6)	χ^2^ = 33.23, *P* < 0.001*
Depression	20 (12.0)	4 (2.4)	*P* < 0.001*^†^
Age (years, mean ± standard deviation)	33.2 ± 12.4)	33.0 ± 12.3)	t(166) = 1.2, *P* = 0.22^‡^
Education level (mean ± standard deviation)	2.2 ± 0.65	2.2 ± 0.65	t(166) = -1.6, *P* = 0.10^‡^
Income level (mean ± standard deviation)	1.8 ± 0.75	2.1 ± 0.74	t(166) = -3.6, *P* < 0.001^‡^
Number of trauma events (mean ± standard deviation)	13.1 ± 8.3	0.6 ± 0.8	t(172) = 19.4, *P* < 0.001^‡^

*McNemar test.

†Binomial distribution used.

‡*t* test.

**Table 2:** Impact of trauma on different life spheres in displaced war survivors and matched controls

Impact of war on	No. (%) of total participants		Displaced survivors (n = 167) (mean ± standard deviation)	Controls (n = 167) (mean ± standard deviation)	
Family adaptation:			2.6 ± 1.3	0.4 ± 0.9	t (166) = 18.5, *P* < 0.001
no impact	91	33.6			
mild to moderate impact	40	14.8			
marked to severe impact	140	51.6			
Social adaptation:			2.5 ± 1.3	0.5 ± 0.9	t (166) = 16.9, *P* < 0.001
no impact	90	33.2			
mild to moderate impact	63	23.3			
marked to severe impact	118	43.5			
Economic adaptation:			2.8 ± 1.3	0.8 ± 1.2	t (166) = 15.7, *P* < 0.001
no impact	77	28.4			
mild to moderate impact	44	16.2			
marked to severe impact	150	55.4			
Occupational adaptation:			0.8 ± 1.4	0.1 ± 0.6	t (165) = 5.5, *P* < 0.001
no impact	206	76			
mild to moderate impact	22	8.1			
marked to severe impact	43	15.9			
Educational adaptation:			0.7 ± 1.1	0.1 ± 0.4	t (166) = 7.4, *P* < 0.001
no impact	201	74.2			
mild to moderate impact	46	17.0			
marked to severe impact	24	8.8			

### Factors associated with psychiatric outcome

Two logistic regression analyses (method enter) were conducted using the diagnoses of current PTSD and current depression as the dependent variables. The ratio of cases to independent variables was 15.6 to 1, which was satisfactory. In the analysis where current PTSD was used as the dependent variable (Table 3), the overall prediction model was reliable (χ^2^ = 72.0, df = 1, 11, *P* < 0.001) and explained 52.0% of the variance (Nagelkerke R^2^). PTSD significantly related to old age, male sex, high levels of perceived distress during exposure to traumatic stressors, and negative cumulative impact of trauma on life spheres. When the logistic regression analysis was repeated with current major depressive episode as the dependent variable (Table 4), the overall model was again significant (χ^2^ = 32.6, df = 1, 11, *P* < 0.001) and explained 32.2% of the variance (Nagelkerke R^2^). Only high levels of distress during exposure to war stressors related to current depression. Interestingly, there was no significant association between the impact of trauma on economic, social, family, occupational, and educational adaptation and depression.

**Table 3:** Factors associated with posttraumatic stress disorder in displaced war survivors persons (n = 173)

Predictors	Beta	Standard error	Wald test	Odds ratio (95% confidence interval)	*P*
Age	0.06	0.02	5.183	1.06 (1.01-1.11)	0.02
Sex (male)	-1.51	0.53	8.192	0.22 (0.08-0.62)	0.004
Marital status	-0.16	0.60	0.071	0.85 (0.26-2.77)	0.79
Education	-0.19	0.39	0.240	0.83 (0.38-1.78)	0.62
Level of income	-0.48	0.37	1.657	0.62 (0.30-1.29)	0.20
History of psychiatric illness	1.07	1.56	0.475	2.93 (0.14-62.34)	0.49
Family history of psychiatric illness	-1.01	0.67	2.259	0.37 (0.10-1.36)	0.13
Time since trauma	-0.001	0.01	0.005	0.99 (0.98-1.02)	0.94
Distress during trauma	1.15	0.46	6.145	3.15 (1.27-7.81)	0.013
Loss of control during trauma	-0.07	0.34	0.048	0.93 (0.48-1.80)	0.83
Global impact of trauma on life	0.18	0.08	4.400	1.19 (1.01-1.41)	0.04

**Table 4:** Factors associated with depression in displaced war survivors (n = 173)

Predictors	Beta	Standard error	Wald test	Odds ratio (95% confidence interval)	*P*
Age	0.03	0.03	1.344	1.03 (0.98-1.09)	0.25
Sex (male)	-0.86	0.61	1.962	0.42 (0.13-1.41)	0.16
Marital status	0.19	0.64	0.093	1.22 (0.35-4.28)	0.76
Education	-0.24	0.45	0.27	0.79 (0.32-1.92)	0.60
Level of income	-0.42	0.42	0.972	0.66 (0.29-1.51)	0.32
History of psychiatric illness	-0.07	1.44	0.002	0.93 (0.06-15.62)	0.96
Family history of psychiatric illness	-1.12	0.81	1.931	0.33 (0.07-1.59)	0.17
Time since trauma	0.01	0.01	0.787	1.01 (0.99-1.04)	0.38
Distress during trauma	1.22	0.50	6.227	3.41 (1.30-8.92)	0.013
Loss of control during trauma	-0.42	0.36	1.413	0.66 (0.33-1.32)	0.24
Global impact of trauma on life	-0.004	0.09	0.002	0.99 (0.84-1.19)	0.96

## Discussion

Comparisons between displaced war survivors and control participants matched on age, sex, and education revealed significant differences in terms of impact of war stressors on family, occupational, social, economic, and educational adaptation. This finding supported our first hypothesis that refugees and internally displaced persons from war settings endure socioeconomic disadvantage, problems in family functioning, lack of access to educational and occupational opportunities, loss of social support, and perhaps marginalization and isolation. It needs to be noted, however, that it is not possible to attribute this negative impact on life domains to displacement only, because our study participants had also been exposed to multiple war-related traumatic events, which may have had independent effects on adaptation.

The strongest predictor of PTSD and depression in internally displaced persons and refugees was the level of perceived distress during exposure to war-related stressors This finding supported our second study hypothesis and is consistent with previous studies which showed that perceived stressfulness of the stressors, rather than mere exposure to them, determines traumatic stress in war and torture survivors (9,16,19,32,33). According to the “learning theory” of traumatic stress, distress, fear, or panic brought about by an inability to escape from the stressor, avoid the occurrence of the event, or reduce its impact, leads to a sense of helplessness with respect to possible future occurrences of the event, thereby causing anxiety reactions, including PTSD symptoms (34-36). In war settings, exposure to multiple stressor events may reinforce the learned helplessness effects of the primary traumatic event and may lead to more certain helplessness and anxiety reactions (34). Indeed, in studies involving survivors of war (9) and earthquake (37) anticipatory fear of recurrence of trauma events and associated helplessness feelings and cognitions in the aftermath of trauma exposure emerged as the strongest predictor of PTSD.

The significant association between distress during trauma and major depressive episode is consistent with previous similar association in war (9), torture (18), earthquake (37,38), and tsunami survivors (39). As PTSD and depression are highly comorbid conditions (46% of survivors with PTSD also had depression in this study), it is possible that they share the same mediating factors and depression develops secondary to PTSD (38,39). Such secondary depression might be related to the helplessness- and hopelessness-generating effects of trauma exposure and subsequent traumatic stress problems. Exposure to multiple war stressors may also lead to hopelessness and depression.

Global impact of trauma on adaptation in different life domains showed independent but rather weak association with PTSD and it was not associated with depression, a finding that provided only partial support for our third study hypothesis. The mechanisms by which such secondary effects of trauma have an influence on PTSD symptoms are not entirely clear. On the one hand, psychological trauma could be conceptualized as involving exposure to primary traumatic events which then lead to secondary stressors, all contributing to an ongoing traumatization process (5,15,40-42). For example, loss of economic opportunities and lower income may be a direct result of the primary trauma (eg, exposure to a war-related traumatic stressor) because the latter may be associated with loss of bread-winning family members, medical problems due to torture or serious physical injury, or sudden loss of property. They may thus act as constant reminders of the primary traumatic events and aggravate PTSD symptoms. It is also possible that such secondary stressors reinforce the helplessness effects of the primary trauma. On the other hand, given that PTSD symptoms lead to serious disability in social, occupational, and family functioning, the psychological reactions in response to secondary effects of trauma on life spheres might be the consequence rather than the cause of PTSD. Although attempt was made to rule out the latter effect in assessments, the retrospective nature of the study does not preclude such confounding.

The findings need to be interpreted in the light of some methodological limitations. Although the sample size afforded sufficient power for the analyses it was still small and findings from such small samples may have limited generalizability. However, the fact that the predictors were consistent with previous research supports the validity of the findings. Furthermore, although measures were taken to minimize sampling bias, non-random selection of study participants may have resulted in a sample with limited representativeness of displaced war survivors in Croatia. Finally, the retrospective design of the study, which involved recall of events and associated reactions assessed on average nine years post-trauma may have affected the internal validity.

In conclusion, our findings suggest that psychological processes during exposure to war stressors are the most important determinants of PTSD and depression in displaced war survivors. Although adaptation in different life spheres after trauma exposure may have an impact on mental health, its effect is not robust and consistent across disorders. These findings imply that a non-trauma-focused approach in rehabilitation of displaced war survivors is not justified. Trauma-focused cognitive-behavioral treatments and exposure-based interventions have been shown to be effective in a wide range of trauma survivors (43). Given the critical role played by distress, fear, and helplessness responses in posttraumatic stress, restoration of sense of control over fear would be effective in recovery from traumatic stress in war survivors. Indeed, treatment studies have found that a behavioral intervention designed to enhance sense of control over fear is highly effective in reducing PTSD and depression in natural disaster survivors (38,44,45) and traumatized refugees (33,34,46). More studies are needed to test the efficacy of trauma-focused interventions in refugees and internally displaced persons.
